# Immersion-Controlled Nanoparticle Electrodeposition on a Patterned Pyrolyzed Photoresist Film Electrode for Single-Entity Electrochemistry Studies Using Scanning Electrochemical Cell Microscopy

**DOI:** 10.1021/acs.langmuir.6c03912

**Published:** 2026-09-28

**Authors:** Takashi Ito, Kathleen O. Bailey, Josselyn De Leon, Naomi F. Harvey, Lingjie Zhang, Lane A. Baker

**Affiliations:** † Department of Chemistry, 5308 Kansas State University, Manhattan, Kansas 66506-0401, United States; ‡ Department of Chemistry, 14736 Texas A&M University, College Station, Texas 77843, United States

## Abstract

We report immersion-controlled electrodeposition as a simple means to fabricate a substrate with defined zones of differently sized nanoparticles and demonstrate its application in scanning electrochemical cell microscopy (SECCM) studies. Defined zones were formed via electrodeposition accompanied by stepwise increases in the immersion depth of a vertically oriented substrate electrode. In this study, the method was demonstrated for the cathodic deposition of platinum nanoparticles (PtNPs) on a pyrolyzed photoresist film (PPF) electrode with a comb-shaped pattern. The sizes and surface immobilization density of PtNPs were measured by scanning electron microscopy, and currents from the hydrogen evolution reaction (HER) in 0.1 M HClO_4_ were measured in each zone by SECCM. The stepwise deposition via cyclic voltammetry enabled reproducible formation of distinct zones decorated with well-separated PtNPs of controlled sizes (10–50 nm in radius). The resulting PPF substrate was suitable for single-entity SECCM measurements, showing HER current proportional to the number of PtNPs in an SECCM droplet and a correlation between HER activity and PtNP size.

## Graphical abstract

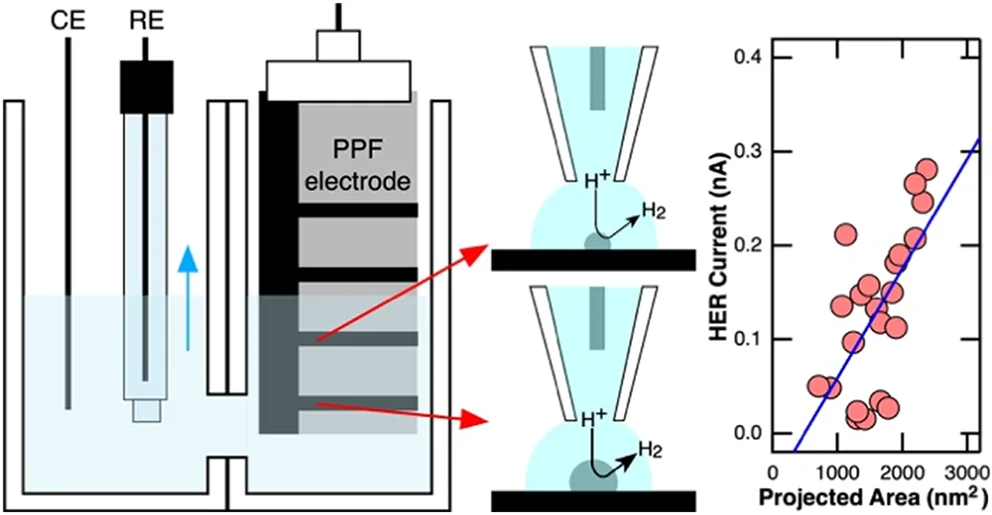

## Introduction

Electrode modification with metal nanoparticles (NPs) has proven to be a widely adopted strategy for studies of electrocatalytic materials for conversion of naturally abundant molecules (e.g., water and CO_2_) to valuable chemicals.^1–3^ The electrocatalytic activities of NP-modified electrodes are routinely assessed from onset potential, turnover frequency, Faradaic efficiency, and/or a Tafel plot upon normalization of the parameters by electrochemical surface area.^4–6^ However, these parameters are traditionally measured for an electrode decorated with a large number of NPs, often with significant heterogeneity in properties such as size, shape, exposed crystal faces, elemental composition, and electrical contact to an underlying electrode.^6–8^ Often, traditional ensemble-averaged measurements cannot discern electrode heterogeneity in the electrocatalytic performance of a NP-modified surface.^6^ Thus, a number of methods, including electrochemical measurements at nanoelectrodes modified with a single NP^9^ and single NP collision experiments,^10^ have been explored to measure the intrinsic electrocatalytic activities of individual NPs.^3,6^

Among these methods, scanning electrochemical cell microscopy (SECCM)^11^ has proven to be a unique technique for measurement of the electrocatalytic activities of single NPs immobilized on conducting substrates.^12–14^ In SECCM, electrochemical measurements are carried out in micro- or nanoscopic regions of a conductive surface, as defined by the intermittent contact of a small droplet formed at the end of a sharp, electrode-incorporating pipette. By scanning the pipette over a surface of interest, electrochemical data at each droplet-surface contact position can be determined to produce an electrochemical activity map of the surface.^12,13^ Electrochemical measurements of single NPs are possible when a single NP is located in an electrochemical cell formed by a solution droplet, which can be attained by the rational control of a pipette opening size (i.e., droplet size) and NP surface coverage.^15,16^ So far, SECCM has been used to assess the electrochemical properties of single NPs in hydrogen evolution reaction (HER),^15,17–20^ oxygen reduction,^18,21^ CO_2_ reduction,^22^ oxygen evolution reaction (OER),^23–25^ hydrazine oxidation,^16,26,27^ borohydride oxidation,^8,28^ and K^+^ insertion and H_2_O_2_ reduction.^29^ Importantly, droplet footprints observed with scanning electron microscopy (SEM) permit the identification of NPs involved in the SECCM measurements.^16^ Correlations between droplet footprints and SECCM data have clarified the effects of NP number,^23^ size,^20,21,29^ and morphology^18,22,25,27^ on electrochemical activities at the single NP level. Such SECCM/SEM measurements can be facilitated by using a substrate electrode that has multiple zones decorated with NPs of different sizes, shapes, or surface coverages. In previous reports, a substrate electrode having multiple zones with distinct surface NP coverages was prepared for SECCM measurements.^23^ The zones were formed by controlling the incubation time for the adsorption of presynthesized nanoscale crystals of zeolitic imidazolate framework-67 (ZIF-67)^23^ via the stepwise control of the immersion depth of a vertically oriented substrate electrode.^30^ SECCM data obtained at the resulting electrode showed a good correlation between the number of ZIF-67 nanocrystals and OER current.^23^ However, there is no prior study demonstrating the fabrication of an electrode with multiple zones based on different-sized NPs for investigating a relationship between the NP size and electrocatalytic activities using SECCM.

In this study, we demonstrate stepwise NP electrodeposition for the fabrication of a substrate electrode with multiple zones that is applicable to single-entity electrochemistry studies using SECCM. Platinum NPs (PtNPs) were cathodically deposited on a pyrolyzed photoresist film (PPF) electrode^31,32^ having a comb-shaped pattern (Figure 1a) that was vertically immersed in a precursor solution (Figure 1b). The immersion depth of the electrode was changed by raising the solution level in a stepwise manner (Figure 1b), which varied the effective electrodeposition time at different zones on the electrode, leading to the formation of PtNPs having different sizes in defined zones. The use of electrodeposition is anticipated to afford NPs with more consistent electrical contact to an underlying electrode as compared with drop-cast NPs.^8^ Here, cyclic voltammetric deposition,^33^ rather than more common galvanostatic step,^21^ potential step,^34,35^ or pulsed potential,^32,36,37^ was employed, which afforded direct monitoring of the electrodeposition process.^38^ Herein, we demonstrate that the immersion-controlled electrodeposition method provides a simple means to prepare a PPF electrode comprising zones decorated with different-sized NPs at a surface density suitable for single-entity electrocatalysis measurements with SECCM.

**Figure 1:**
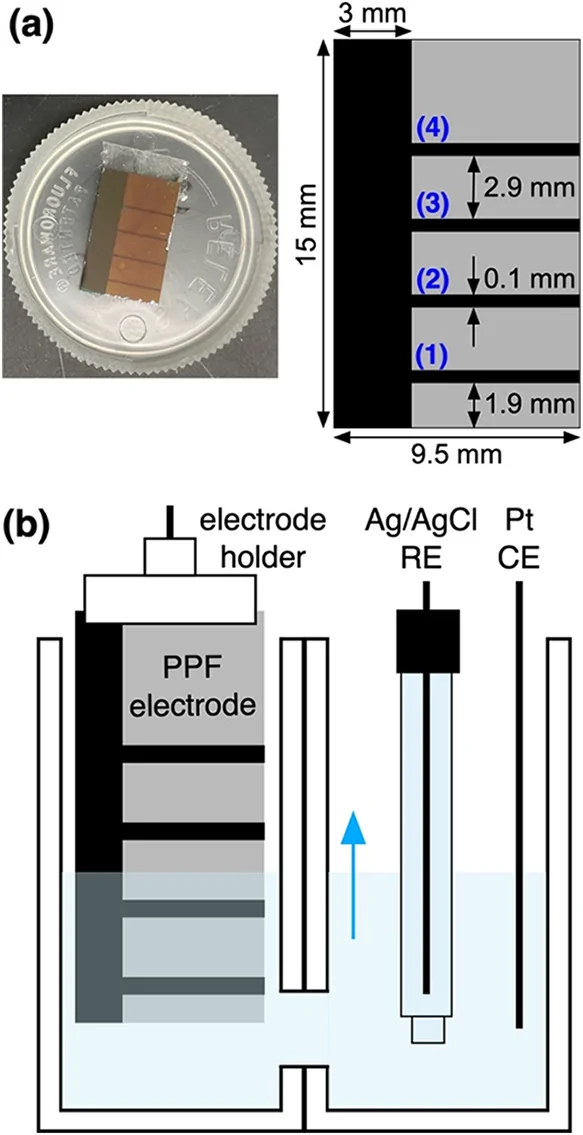
(a) Comb-patterned PPF electrode (left) and its design with the numbering of each band (right). (b) Experimental setup for immersion-controlled electrodeposition on a comb-patterned PPF electrode. The solution level was raised in a stepwise manner to cover one band for each of the deposition steps, to generate a range of electrodeposition times.

## Material and Methods

### Chemicals and Materials

Aqueous chloroplatinic acid solution (8 wt %; H_2_PtCl_6_; Aldrich), concentrated hydrochloric acid (HCl; Fisher), perchloric acid (60%; Sigma-Aldrich), and potassium chloride (Fisher) were used as received. A platinum wire (50 μm in diameter) was purchased from California Fine Wire Co. All aqueous solutions were prepared with water having a resistivity of 18 MΩ·cm (Barnstead Nanopure Systems or Barnstead Genpure Pro).

Silicon (100) wafers (100 mm diameter, p-type, resistivity 1–10 Ω·cm) with a thermal oxide layer were obtained from UniversityWafer, Inc. Nanostrip cleaning solution, MCC adhesion promoter (MicroChem Corp., now part of Kayaku Advanced Materials), Microposit S1813 positive photoresist (DOW), and MF-319 developer (Rohm and Haas Electronic Materials) were used as received without further steps. Deionized water (WaterTech Services) and nitrogen gas (Linde) were used for substrate rinsing and drying, respectively. Forming gas consisting of 5% H_2_ and 95% N_2_ (Linde) was used during the pyrolysis process.

### Fabrication of PPF Electrodes

PPF electrodes were prepared according to a previously reported procedure^32^ with modifications. Silicon wafers were first cleaned by soaking in Nanostrip solution (Caution: Nanostrip is a strong oxidizing agent) for at least 30 min. The wafers were then rinsed thoroughly with deionized water, dried under a stream of nitrogen, and baked on a hot plate at 250 °C for 5 min. After cooling, cleaned substrates were spin-coated (Laurell PHOTO Spin Coater) with an MCC adhesion promoter and were soft-baked at 115 °C for 1 min. Microposit S1813 positive photoresist was then spin-coated using a two-step program on a spin coater: (1) 500 rpm (acceleration 100 rpm/s) for 15 s; (2) 2500 rpm (acceleration 300 rpm/s) for 45 s. A second soft bake was performed at 115 °C for 1 min. Photoresist films were subsequently exposed using maskless lithography (Heidelberg MLA 150 Maskless Lithography System) with Klayout software at a dose of 150 mJ/cm^2^ (defocus 0). Following exposure, the substrates were developed in MF-319 developer for 1 min to remove the exposed photoresist, followed by rinsing with deionized water and drying under a stream of nitrogen. The final soft bake was conducted at 115 °C for 1 min. Patterned substrates were pyrolyzed in a tube furnace (MINIBRUTE Oxidation/Anneal Furnace) under a forming gas atmosphere (5% H_2_, 95% N_2_). The furnace temperature was ramped at 5 °C/min to 1000 °C (with total heating and cooling times of approximately 3 h 20 min each) and held at 1000 °C for 1 h to yield conductive pyrolyzed photoresist film (PPF) electrodes. Individual electrode pieces (approximately 15 mm × 9.5 mm) were obtained by dicing the wafer using a Disco dicing saw (DISCO Corporation).

Of note, a conductive carbon layer was present not only on the four bands and backbone of the comb pattern but also at interband regions owing to incomplete removal of a photoresist layer during the developing process, as indicated by their color/contrast in the optical and SEM images different from the exposed SiO_2_ surface (Figure S1a–c). Thus, PtNPs of similar sizes and surface density were deposited on the band, backbone, and interband regions of each zone (Figure S1d).

### Immersion-Controlled Electrodeposition of PtNPs

A cell having two reservoirs (Figure 1b) was prepared from two plastic cuvettes (Perfector Scientific) adhered by epoxy (Loctite Hysol 1C) and connected by a through-hole (*ca*. 5 mm in diameter). A PPF comb electrode was vertically immersed in an aqueous solution containing 1 mM H_2_PtCl_6_ and 0.1 M HCl^35^ using a working electrode holder (StonyLab) (Figure 1b). PtNPs were electrodeposited on the PPF electrode by cycling a potential between +0.5 and −0.3 V (vs Ag/AgCl, with *iR* compensation) 50–200 times in a three-electrode cell consisting of a Ag/AgCl reference electrode (with 3 M KCl) and a platinum wire counter electrode using a CH Instruments Model 660B electrochemical analyzer. *iR* compensation was used because the deposition took place with a relatively large cathodic current (0.5–1.5 mA; see Figure 2) at the PPF electrodes with relatively high resistance (300–500 Ω). The immersion depth of the PPF electrode was increased from one band to the next by adding the 1 mM H_2_PtCl_6_ solution to the reservoir with the reference and counter electrodes after each electrodeposition step. The electrodeposited PPF electrode was washed with water, dried by a nitrogen stream, and then used for SECCM and SEM measurements without any further treatments.

**Figure 2:**
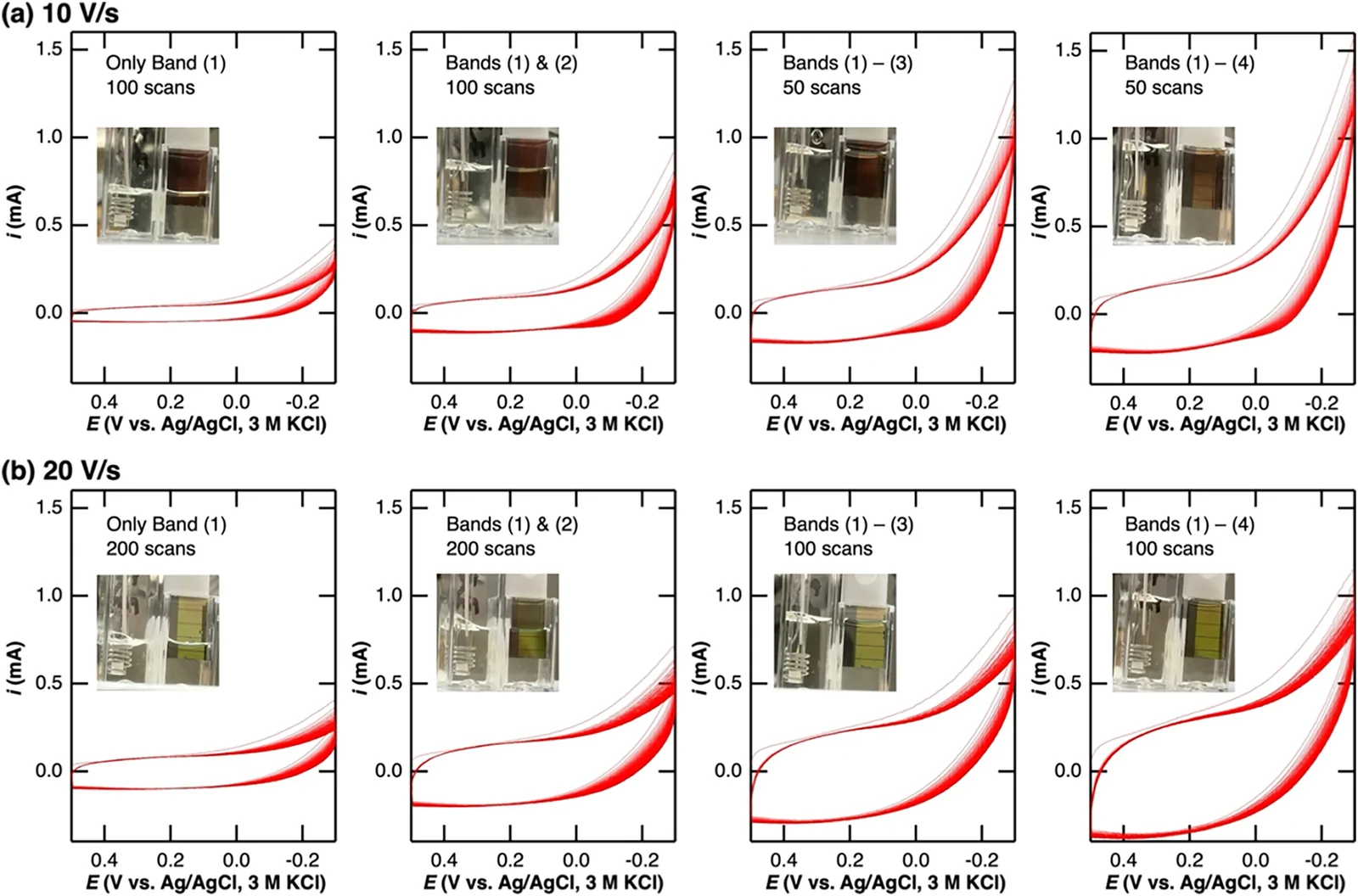
CV data obtained during the zone electrodeposition of PtNPs in 1 mM H_2_PtCl_6_ + 0.1 M HCl at (a) 10 V/s and (b) 20 V/s. The solution level was raised in a stepwise manner to control the immersion depth of a PPF electrode, as shown in the inset pictures.

### Probe Fabrication

Pipettes were fabricated from quartz capillaries (O.D. 1.0 mm, I.D. 0.70 mm, 7.5 cm length; Sutter Instruments) on a p-2000 laser puller (Sutter Instruments). Tips were pulled to approximately *ca*. 0.24 μm in opening radius (Figure S2), using the following parameters: HEAT = 540, FIL = 3, VEL = 40, DEL = 190, PUL = 180.

### SECCM Measurements

SECCM cyclic voltammetry (CV) measurements were performed in the hopping mode^12^ on a home-built instrument, using a two-electrode system.^39^ A Ag/AgCl quasi-reference-counter electrode (QRCE) was used. QRCE was fabricated by chloridizing a silver wire (A-M Systems) in 1 M FeCl_3_. The open-circuit potential of the QRCE against a purchased reference electrode (CH Instruments, Inc.) was measured in 0.1 M perchloric acid (potentiostat: CH Instruments, Inc., CHI660C) and resulted in +0.298 V (vs Ag/AgCl, 3 M KCl). The QRCE was inserted into a pipette filled with 0.1 M perchloric acid solution. A PPF electrode sample was housed in a 3D-printed environmental chamber under a background of flowing Argon gas. Digital cameras were used to aid in pipette positioning. Coarse pipette position was achieved using a XYZ stepper motor, and piezoelectric positioners were used for fine movement and approach to the substrate. A bias of −1 V (vs Ag/AgCl QRCE) was applied during approach. When the pipette meniscus contacted the substrate, the current feedback was used to halt the approach. After establishing contact, the pipette was offset to begin mapping. The pipette approached the substrate at a rate of 3 μm/s and maintained contact at the surface to sweep the potential. The pipette was then retracted 10 μm, moved 4 μm in the *y*-direction, and reapproached, with this cycle repeated to complete an electrochemical map. CV measurements were swept at a rate of 1 V/s, from 0 to −1.2 V (vs Ag/AgCl QRCE). A fresh pipette was used to measure SECCM data at each zone, because a pipette used for many measurements afforded a small cathodic current attributed to HER caused by contaminants in the droplet solution (Figure S3). Resulting SECCM maps had a hopping distance of 4 μm between pixels. SECCM current was measured using a current amplifier (VA-10). Instrumentation was controlled by a custom program in a LabVIEW interface using a field-programmable gate array (FPGA). SECCM data were analyzed using Python and Igor Pro 9.

### SEM Measurements

PPF electrodes decorated with PtNPs before and after the SECCM measurements were measured using a Hitachi S-4700-II SEM at the Center of Nanoscale Materials, Argonne National Laboratory, at an acceleration voltage of 1–15 kV. SEM images were analyzed using ImageJ software.^40^ The radius of each PtNP was estimated from its area, assuming a spherical particle.

## Results and Discussion

In this study, we explored the immersion-controlled electrodeposition method (Figure 1) to fabricate multiple distinct areas decorated with different-sized PtNPs on a substrate electrode for single-entity electrocatalysis measurements with SECCM. The resulting electrode permits a series of SECCM experiments under varying electrodeposition times to be carried out without electrode replacement. First, we verified electrodeposition at specific zones by measuring the size and surface immobilization density of PtNPs using SEM. Subsequently, we conducted SECCM measurements to assess the effects of nanoparticle size on HER current in an ensemble-averaged manner. Finally, we discuss the correlation of HER current with the number of measured PtNPs, and then a size–activity relationship from SECCM data obtained for single PtNPs.

### Immersion-Controlled PtNP Electrodeposition on PFF Electrodes

Figure 2 shows CV data recorded during the 4-step immersion-controlled electrodeposition of PtNPs on a comb-patterned PPF electrode (Figure 1a) at (a) 10 V/s and (b) 20 V/s. The solution level was raised after each CV-based deposition step, as shown in the inset pictures, leading to the observation of a larger charging current ascribed to the deeper immersion of the vertically oriented PPF electrode in solution. Pt deposition was implied by a slight increase in cathodic HER current at *E* < −0.1 V from the 30th scan and also by the growth of an anodic current shoulder around *E* = −0.1 at 10 V/s (Figure S4) that could be attributed to hydrogen desorption from the Pt surface.^38^

Figure 3a shows SEM images of the four bands resulting from the CV-based PtNP deposition. In all cases, surface PtNP density was low (≤2 particles/μm^2^) enough to measure single PtNPs using a droplet formed at the end of a 0.24-μm radius SECCM pipette. Interestingly, PtNP density was higher at 10 V/s than at 20 V/s, and was not significantly altered by the number of CV scans (Figure 3b). These observations suggest that the surface PtNP density was mainly controlled by nucleation kinetics,^33^ owing to the application of a relatively small overpotential for Pt­(IV) reduction (i.e., ca. −0.1 V vs SHE at the most negative applied potential in the CV-based deposition) for a short period of time associated with the fast scan rate. On the other hand, PtNP radius increased with the number of CV scans (Figure 3c), enabling the deposition of different-sized PtNPs on distinct areas. This observation implies that Pt­(IV) was preferentially reduced on PtNPs formed during the initial deposition step, rather than on the PPF surface. Importantly, the immersion-controlled electrodeposition method was reproducible with tolerable size distributions, as indicated by the formation of PtNPs of similar sizes and surface immobilization density on different PPF electrodes (Figure 3b,c).

**Figure 3:**
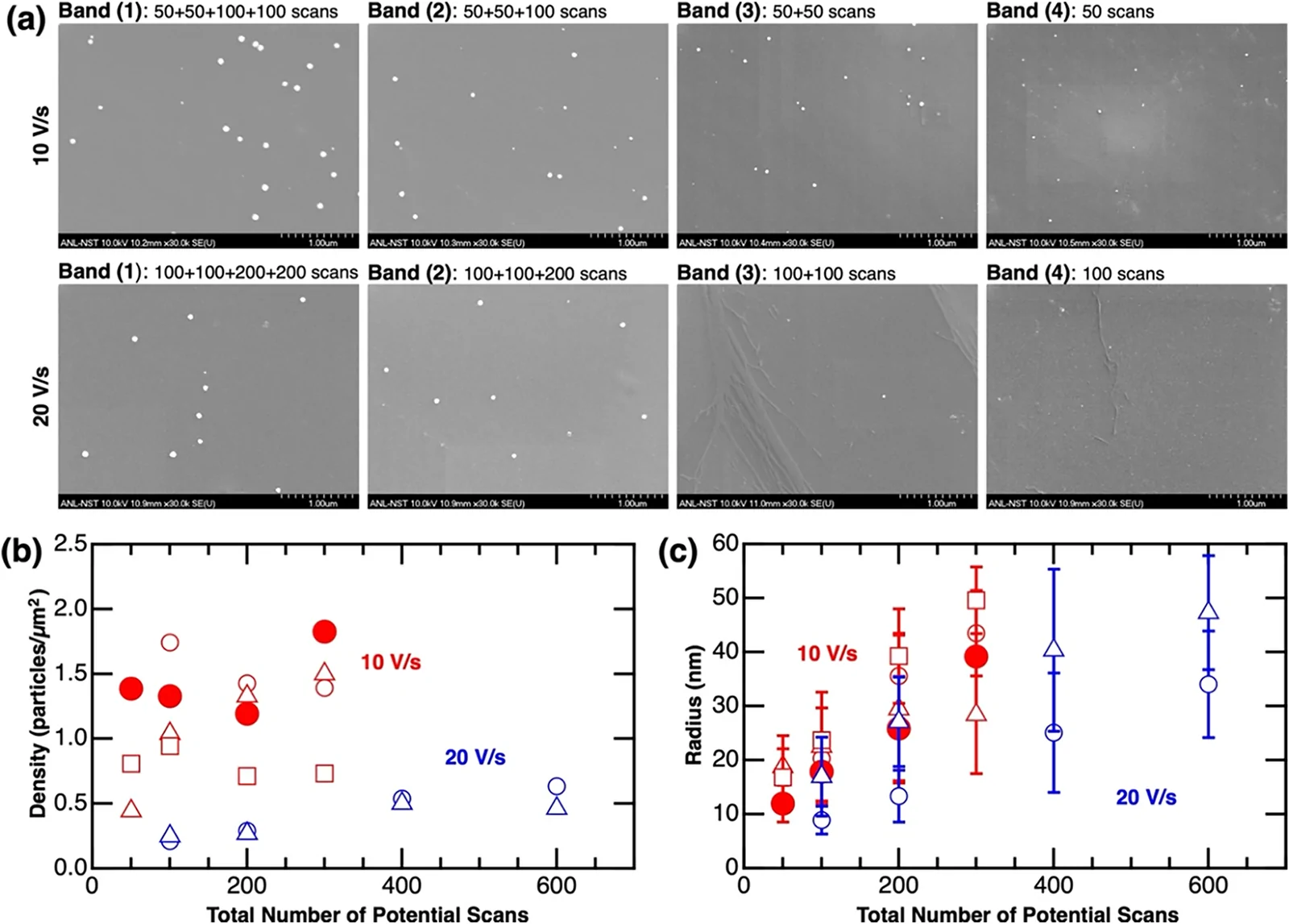
(a) Typical SEM images of the four bands of PPF electrodes decorated with PtNPs at 10 and 20 V/s. Typical CV data recorded for the preparation of these samples are given in Figure 2. (b) Surface density (the number of particles per μm^2^ from multiple SEM images; see Figure S5) and (c) the radius of deposited PtNPs as a function of the total number of CV scans. Different symbols indicate data obtained from PPF electrodes prepared at 10 V/s (in red) and 20 V/s (in blue) on different days, including closed red circles from data obtained at a PPF electrode used for the SECCM measurements (see below). Error bars in (c) depict the standard deviations of radius from multiple PtNPs. The variation in the density and radius obtained on different samples could be attributed to differences in the conductivity and surface properties of the PPF films.

### Ensemble-Averaged Assessment of SECCM Data

SECCM was used to assess the HER activities of four zones on a PPF electrode decorated with PtNP at 10 V/s (Figure 3b,c, closed red circles). Figure 4 shows SECCM-CV data recorded at 96 positions on each of the four zones. Many of the SECCM-CV data showed a cathodic current at ≤−0.5 V (vs Ag/AgCl QRCE), which could be attributed to HER, as supported by SECCM-CV data recorded on a Pt wire (Figure S6). A larger HER current, *i*_HER_ (=*i*_–1.2 V_ – *i*_–0.2 V_, which corresponds to a current after the subtraction of a charging current), was observed at a zone decorated with larger PtNPs, i.e., Band (1) > Band (2) > Band (3) > Band (4). However, *i*_HER_ on each zone varied from position to position, as summarized in Figure 5. Each of the *i*_HER_ histograms consists of two major components: Very small *i*_HER_ (≈0 nA) and larger *i*_HER_, which would reflect the absence and presence of PtNP(s), respectively. The latter was fitted to a Gaussian distribution to determine the ensemble-averaged *i*_HER_ (*i*_HER,ave_) and its distribution (Figure S7a–d). Figure S7e verifies the observation of a larger *i*_HER,ave_ at a zone with larger PtNPs. Unfortunately, this analysis cannot provide a quantitative relationship between PtNP number/size and HER activity owing to the variation of the number and size of PtNPs involved in individual SECCM-CV measurements.

**Figure 4:**
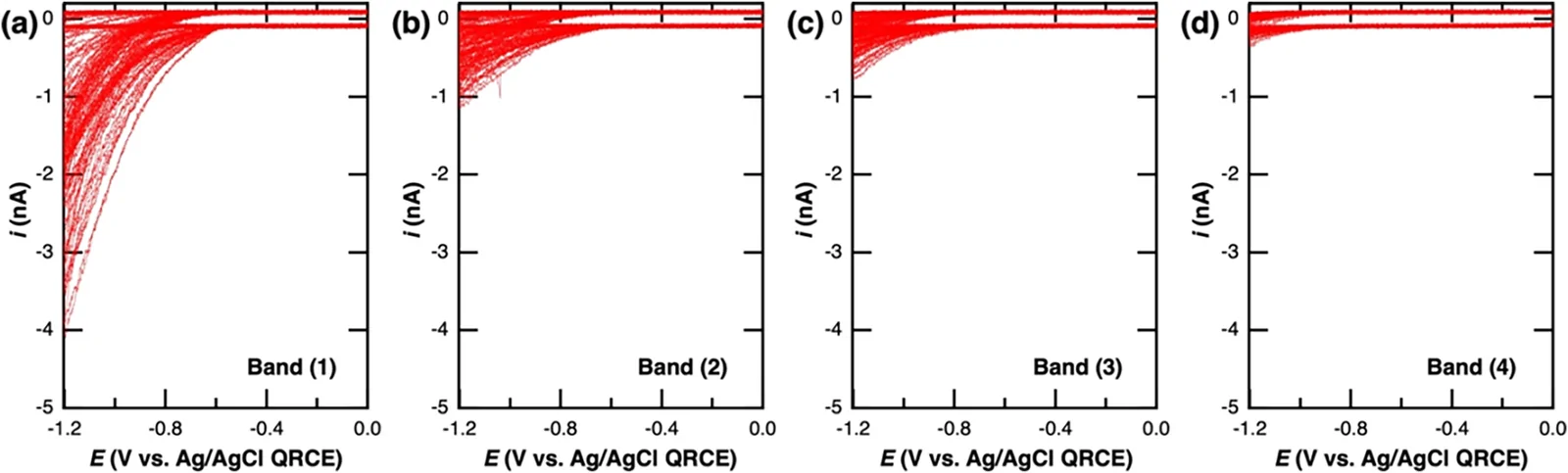
Typical SECCM-CV data (scan rate: 1 V/s; in 0.1 M HClO_4_) obtained at 96 different positions around (a) Band (1), (b) Band (2), (c) Band (3), and (d) Band (4) on a PPF electrode decorated with PtNPs at 10 V/s.

**Figure 5:**
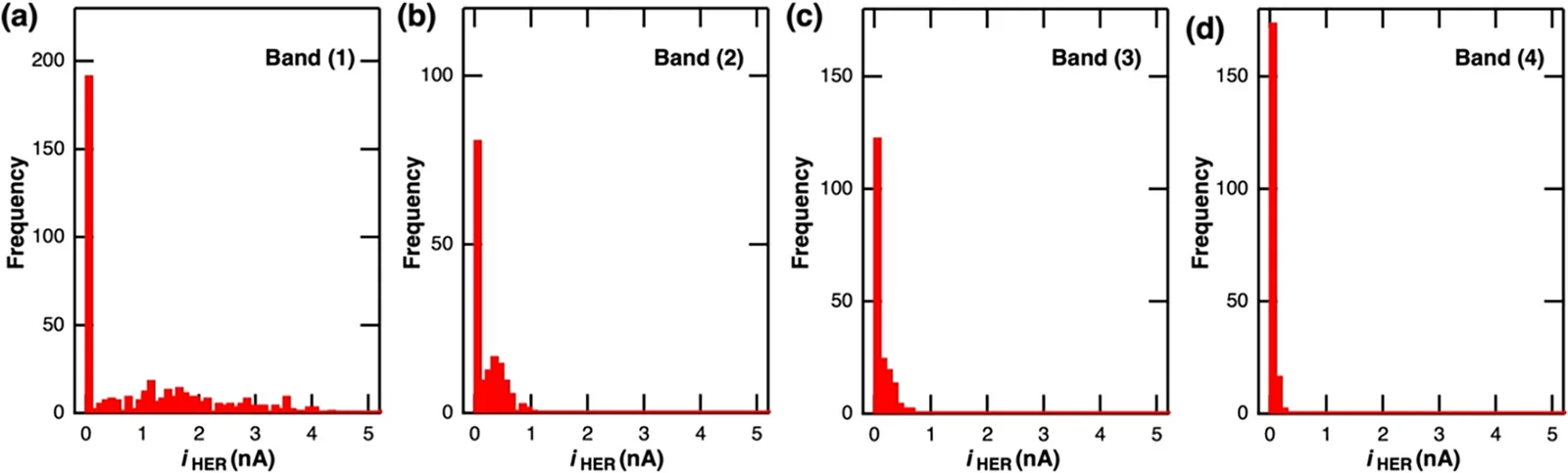
(a–d) Histograms showing the number of observations of *i*_HER_ (=*i*_–1.2 V_ – *i*_–0.2 V_; *i*_–0.2 V_ corresponds to a charging current) obtained at multiple areas of each zone on a PPF electrode decorated with PtNPs at 10 V/s.

### Relationship between PtNP Number/Size and HER Current from SECCM/SEM Data

Relationships between PtNP number/size and HER current could be discussed for SEM/SECCM data obtained at Band (3) that gave clear droplet footprints (Figure S8). Unfortunately, we could not find droplet footprints at Band (1), possibly because of the much higher contrast of PtNPs than footprints in SEM images. Apparent droplet footprints were observed at Band (2) (Figure S9). However, their sizes (ca. 0.1 μm in radius) were smaller than the nominal radius of the pipette opening (*ca*. 0.24 μm), suggesting that the apparent footprints did not reflect droplet-electrode contact areas. While we infer that the apparent footprints observed at Band (2) resulted from landing the pipette on the PPF surface, detailed analysis of SECCM/SEM data was not pursued due to difficulty in determining the measured area from the SEM images.

Figure 6a shows an SECCM map for *i*_–1.2 V_ that was measured around Band (3). The positions of the SECCM-CV measurements could be determined from those of the droplet footprints (Figures S8 and S10), enabling us to directly correlate *i*_HER_ with *N*_PtNP_. The radii of these footprints shown in magnified SEM images (Figure 6b–d) were close to each other (ca. 0.3 μm). The *N*_PtNP_ was in the range of 0–4, which is color-coded in the images, and was correlated with *i*_HER_. Basically, PtNP-free positions gave negligible HER current, and the more PtNPs led to the observation of a larger HER current. The correlation can be verified by plotting *i*_HER_ obtained at all the 96 positions that are shown in Figure 7a. A trend in *N*_PtNP_–*i*_HER_ relationship suggests the stable immobilization and good electrical contact of the electrodeposited PtNPs onto the underlying PPF surface. However, *i*_HER_ values varied for each *N*_PtNP_, which is attributable to the different HER activities of PtNPs having different sizes and shapes/structures^3,8^ and the variation of PtNP position within a droplet.^18^

**Figure 6:**
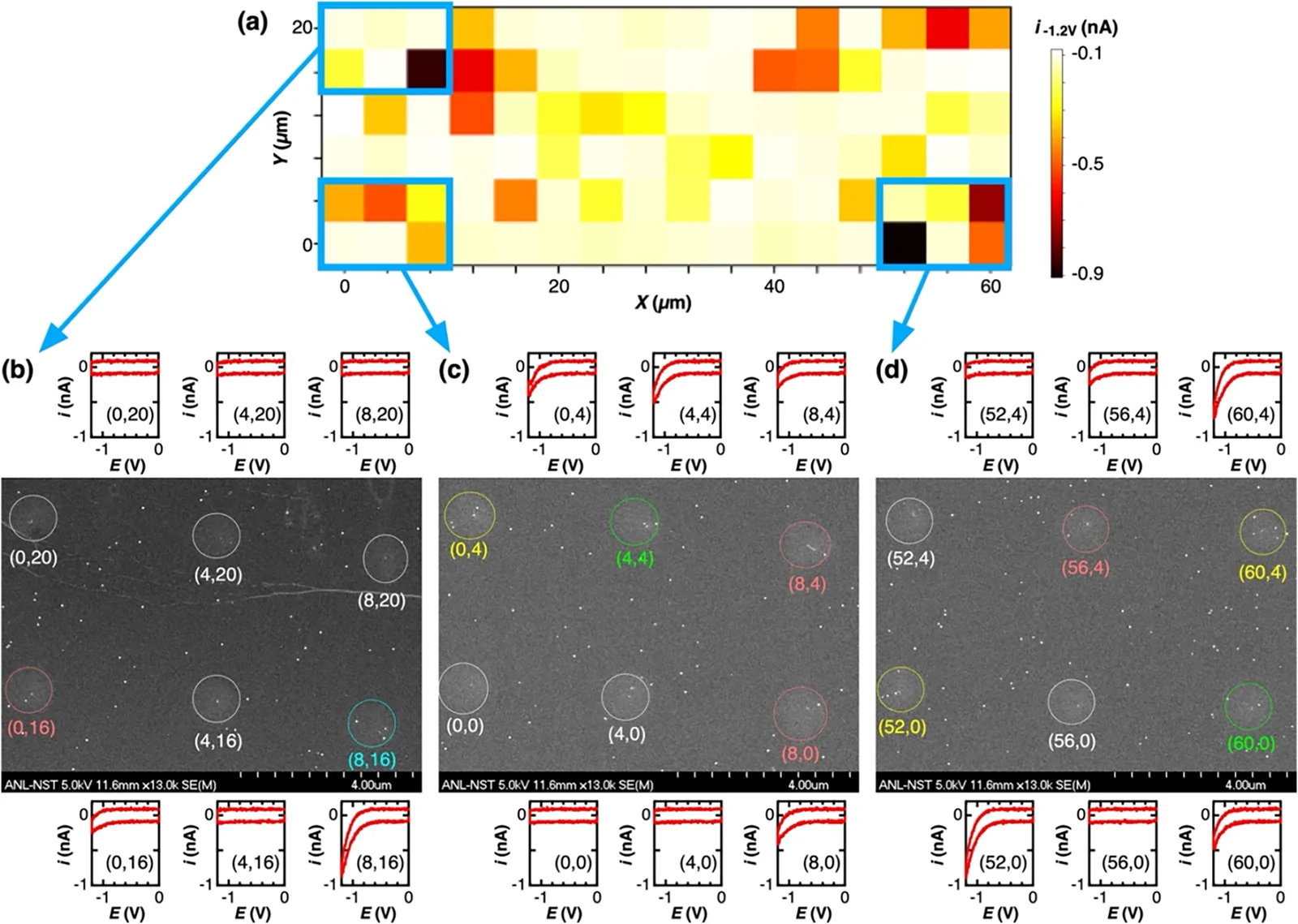
(a) SECCM map based on *i*_–1.2 V_ obtained in 0.1 M HClO_4_ around Band (3) (see Figure S8) on the PPF electrode (10 V/s). (b–d) SEM images at each of the three regions shown by boxes in (a). Droplet footprints are indicated by circles, and SECCM-CV data obtained at each footprint position (scan rate: 1 V/s; *E* (V vs Ag/AgCl QRCE)) are also given. The number of PtNPs in each of the footprints (*N*_PtNP_) was determined by adjusting the contrast of an SEM image as shown in Figure S10.

**Figure 7:**
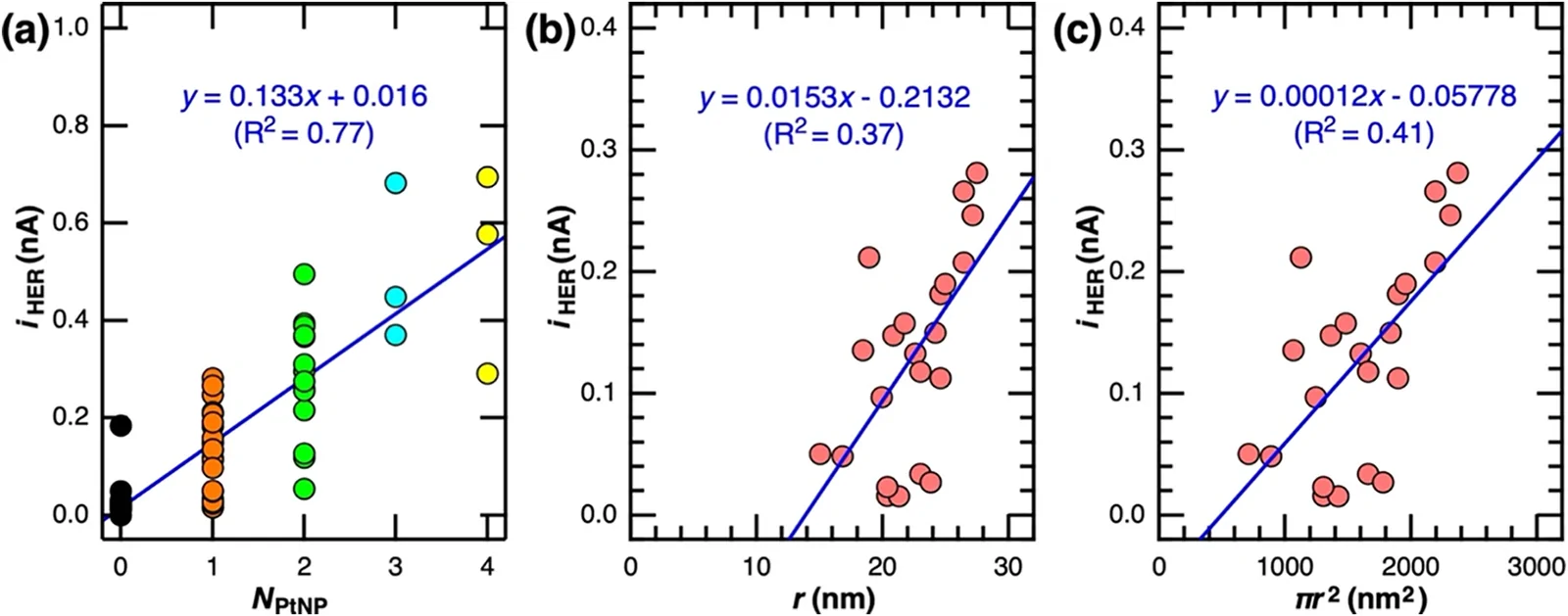
(a) Relationship between *i*_HER_ and *N*_PtNP_. Of note, the colors of the data are consistent with those used in Figure 6b–d. (b, c) Relationship between *i*_HER_ and the radius (*r*) and projected surface area (=π*r* ^2^), respectively, obtained only from footprints with *N*_PtNP_ = 1. Blue solid lines in these graphs were obtained by linear fitting.

Among these possible factors, the effects of PtNP size on *i*_HER_ could be assessed from data obtained only from footprints with *N*_PtNP_ = 1 (Figure 7b,c). The current recorded, *i*_HER_, linearly increased with particle surface area (Figure 7c) rather than particle radius (Figure 7b), though the correlation coefficient was low, probably owing to the significant contributions of the other factors, such as the variation of PtNP position within a droplet^18^ and that of the HER activities of individual PtNPs having different structures including defects.^3,8^ The linear relationship to surface area implies that *i*_HER_ at the large overpotential (ca. 0.7 V) was controlled by linear diffusion of H^+^; This is consistent to the significantly larger diffusion layer thickness of H^+^ at *v* = 1 V/s (ca. 6 μm estimated from $L=\sqrt{\frac{2D_{H^{+}}\mathrm{RT}}{\mathrm{Fv}}}$ ^41^ with *D*_H^+^_ = 7.9 × 10^–6^ cm^2^/s^42^) than the dimensions of the droplet (*ca*. 0.3 μm in radius) and the capillary with a small opening radius (0.24 μm in radius) and a high aspect ratio. The linear relationship between *i*_HER_ and surface area indicates that a current density is independent of PtNP size in this range (10–50 nm in radius), reflecting the quick supply of H^+^, and the fast outgoing mass transport of H_2_ ^43^ in contrast to the size-dependent reduction of ORR current density reported in a prior SECCM study with single electrodeposited silver NPs.^21^

## Conclusions

We successfully demonstrated the fabrication of a substrate electrode comprising four zones decorated with different-sized NPs and their applicability for SECCM measurements. Zones were formed via CV-based electrodeposition accompanied by a stepwise increase in the immersion depth of a vertically oriented PPF electrode by simply raising the solution level. The size of NPs in each zone could be controlled by the total number of potential sweeps, and their surface density could be adjusted by the scan rate. We showed a reasonable correlation between *i*_HER_ and *N*_PtNP_, indicating the stable immobilization and good electrical contact of electrodeposited PtNPs on the PPF electrode. In addition, *i*_HER_ was proportional to PtNP surface area, implying that the HER current was controlled by linear diffusion of H^+^ toward a PtNP within an electrochemical cell defined by the droplet, pipette, and PPF surface. Here, we focused only on CV-based electrodeposition of PtNPs to show the nanoparticle size effect on the HER activity. However, the stepwise control for a vertically immersed electrode will be adaptable for the fabrication of zones comprising NPs of different properties by modulating applied potential form and/or duration. For example, pulsed electrodeposition affords NPs of different shapes with different exposed facets.^44^ The use of pulsed electrodeposition and the immersion-controlled method will thus provide simple means to fabricate zones based on NPs of different structures for single-entity electrocatalysis measurements with SECCM.

## Supplementary Material



## Notes

Optical and SEM images of PPF electrodes, SEM image of an SECCM pipette, histograms on *i*_HER_ obtained using fresh and used pipettes, detailed CV data, SECCM-CV data obtained on a Pt wire, Gaussian curve fitting to *i*_HER_ distribution and a relationship between ensemble-averaged *i*_HER,ave_ and particle projected surface area, and SEM images showing droplet footprints at Band (3) and apparent droplet footprints at Band (2). (PDF)
