# Supplementary material for: Immersion-Controlled Nanoparticle Electrodeposition on a Patterned Pyrolyzed Photoresist Film Electrode for Single-Entity Electrochemistry Studies Using Scanning Electrochemical Cell Microscopy
Source: Langmuir. 2026 Sep 28;42(40):29987–95. doi: 10.1021/acs.langmuir.6c03912 (PMC13674232; doi:10.1021/acs.langmuir.6c03912)
Supplement: Supplementary file 1 [file la6c03912_si_001.pdf]

Supporting Information

Immersion-Controlled Nanoparticle Electrodeposition on a Patterned Pyrolyzed Photoresist Film Electrode for Single-Entity Electrochemistry Studies Using Scanning Electrochemical Cell Microscopy

Takashi Ito,[a,]* Kathleen O. Bailey,[b] Josselyn De Leon,[b] Naomi F. Harvey,[a] Lingjie Zhang,[b] Lane A. Baker[b,]*

[a] Department of Chemistry, Kansas State University, Manhattan, KS 66506-0401, USA

[b] Department of Chemistry, Texas A&M University, College Station, TX 77843, USA

* To whom correspondence should be addressed.

Email: ito@ksu.edu; Phone: 785-532-1451; Fax: 785-532-6666

Email: lane.baker@tamu.edu; Phone: 979-845-4721

Number of Pages: 7

Number of Figures: 10

Table of Contents

Figure S1: Optical and SEM images of a PPF electrode showing uniform PtNP deposition on the PPF surface.
Figure S2: SEM image of the opening of an SECCM pipette.
Figure S3: Histograms on i_{HER} recorded around Band (4) using fresh and pipettes.
Figure S4. CV data during the deposition at Bands (1)–(4) at 10 V/s.
Figure S5: SEM images of the other PtNP-deposited PPF electrodes.
Figure S6: SECCM-CV data obtained on a Pt wire.
Figure S7: Gaussian curve fitting to i_{HER} distribution and a relationship between $i_{HER,ave}$ and projected surface area.
Figure S8: Optical picture of the PPF electrode measured at Band (3) using SECCM and SEM images showing droplet footprints.
Figure S9: SEM images of apparent droplet footprints obtained at Band (2) of the PPF electrode.
Figure S10. SEM images that explain how to determine N_{PtNP}.

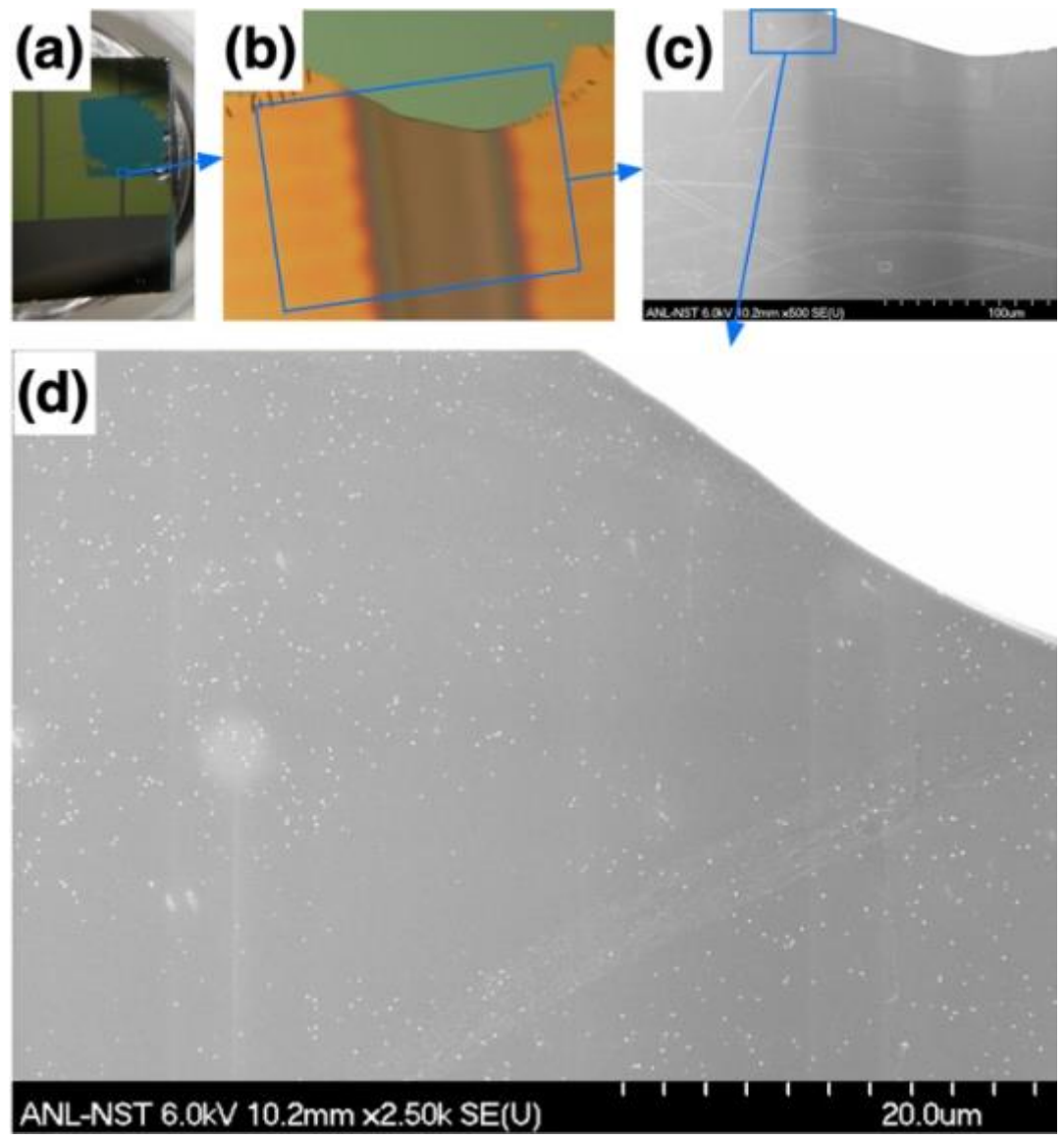

Figure S1. (a,b) Optical microscopic and (c,d) SEM images of a PPF electrode around Band (1) upon 4-times PtNP deposition (*i.e.*, 200 + 200 + 100 + 100 cyclic voltammetric scans in total) at 20 V/s. **Figure S1d** shows that PtNPs of similar sizes were deposited on the band and inter-band regions at a similar density, indicating the presence of a conductive carbon layer on the both regions. The presence of the conductive carbon layer was highlighted by detachment with a tape, exposing underlying oxidized Si that was observed in green in **Figure S1b** and in white (owing to charging) in **Figure S1cd**.

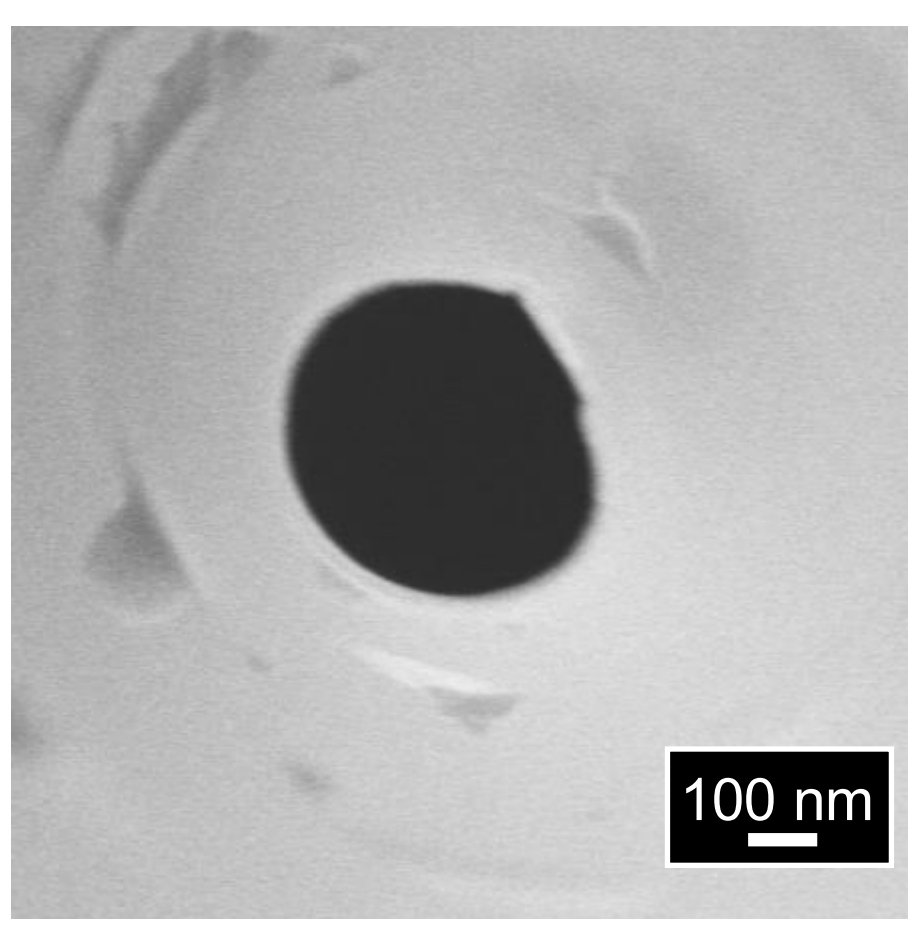

Figure S2. SEM image of a typical SECCM capillary, showing the opening radius of *ca*. 0.24 µm.

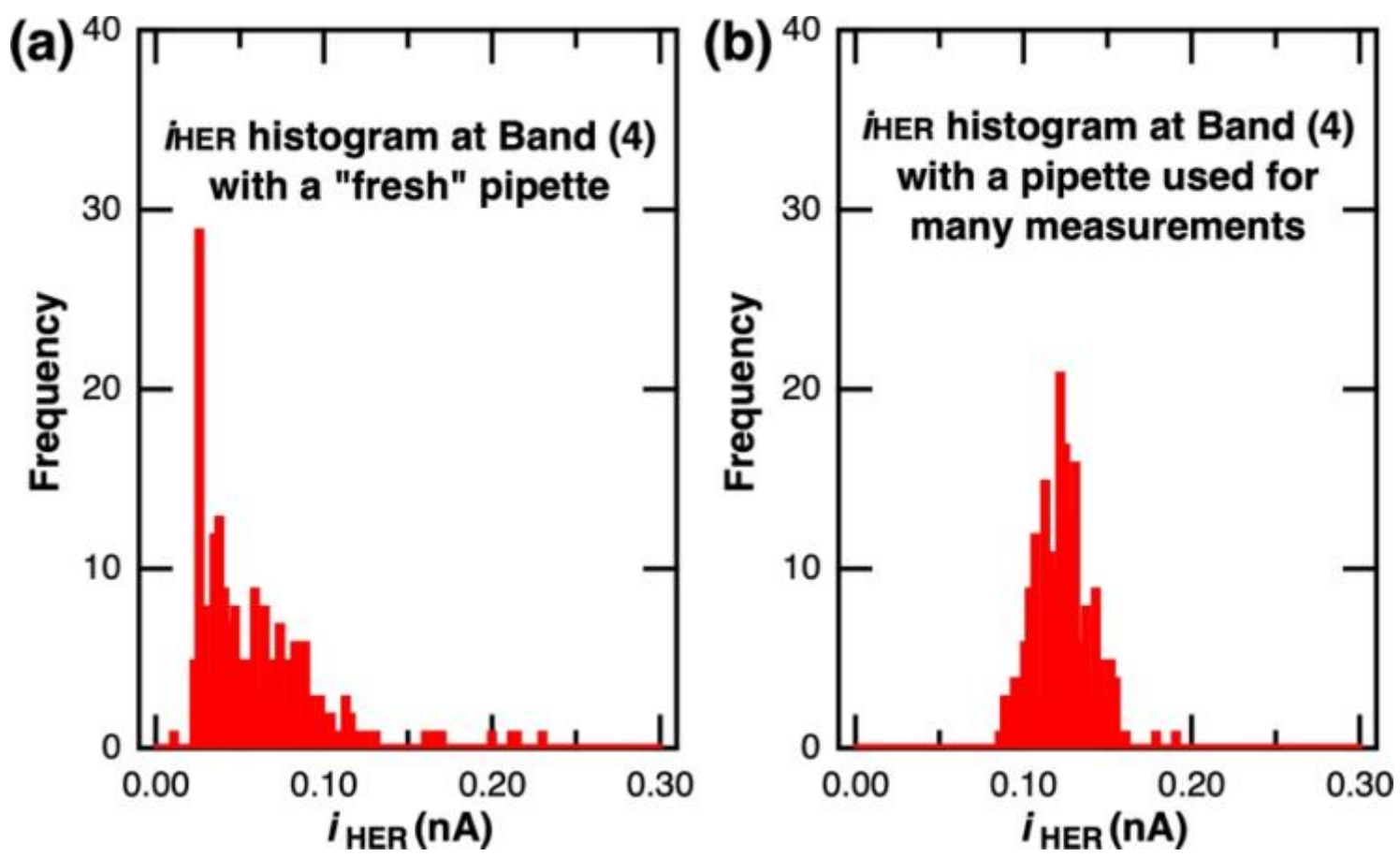

Figure S3. Histograms on i_{HER} recorded around Band (4) on the PPF electrode (10 V/s) obtained using (a) a fresh pipette with no prior SECCM measurement and (b) a pipette that was used for SECCM measurements at Bands (1), (2), and (3).

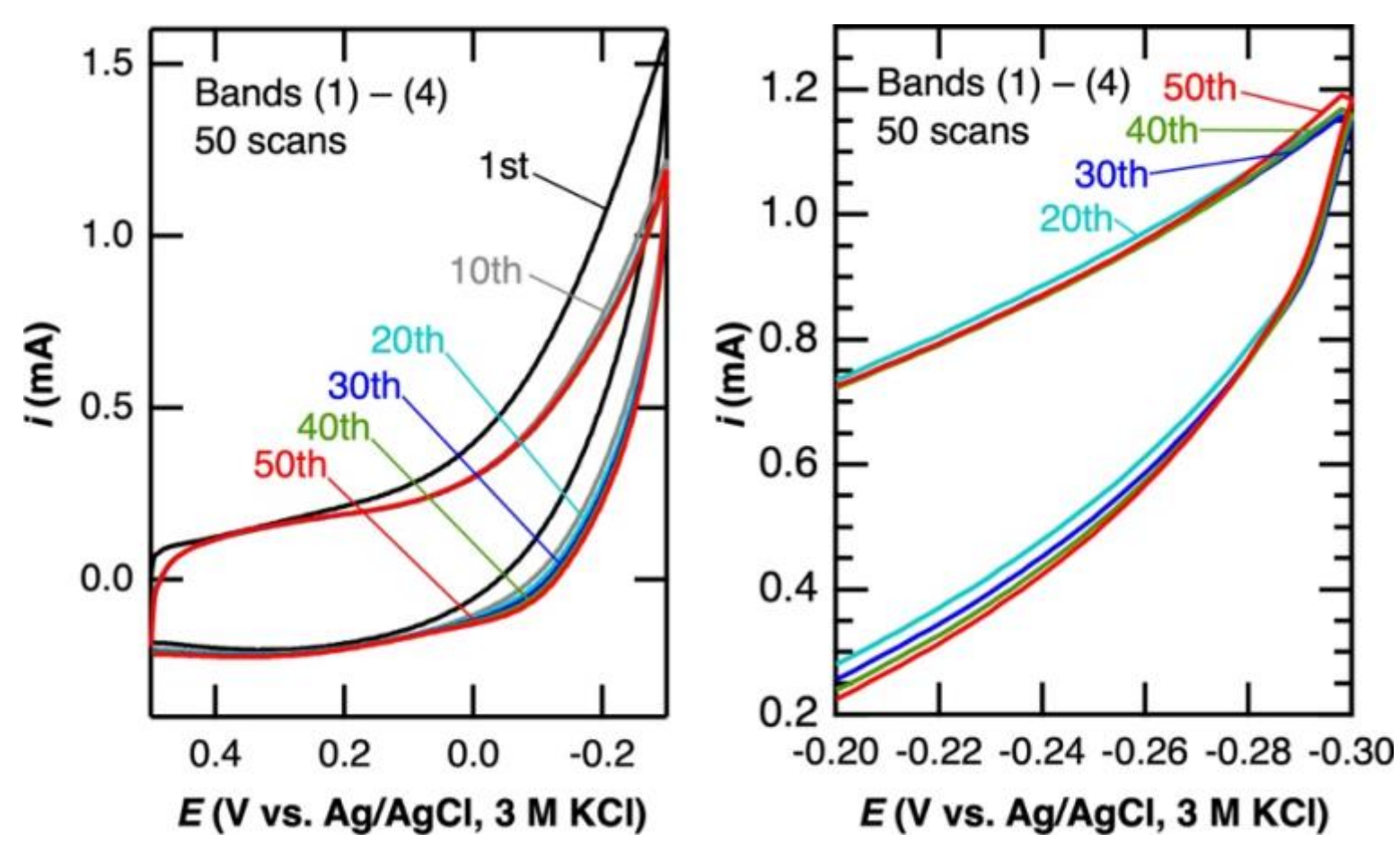

Figure S4. CV data (1st, 10th, 20th, 30th, 40th, and 50th scans) shown in **Figure 2a** (right), obtained during the electrodeposition of PtNPs on Bands (1)–(4) in 1 mM H_2PtCl_6 + 0.1 M HCl at 10 V/s.

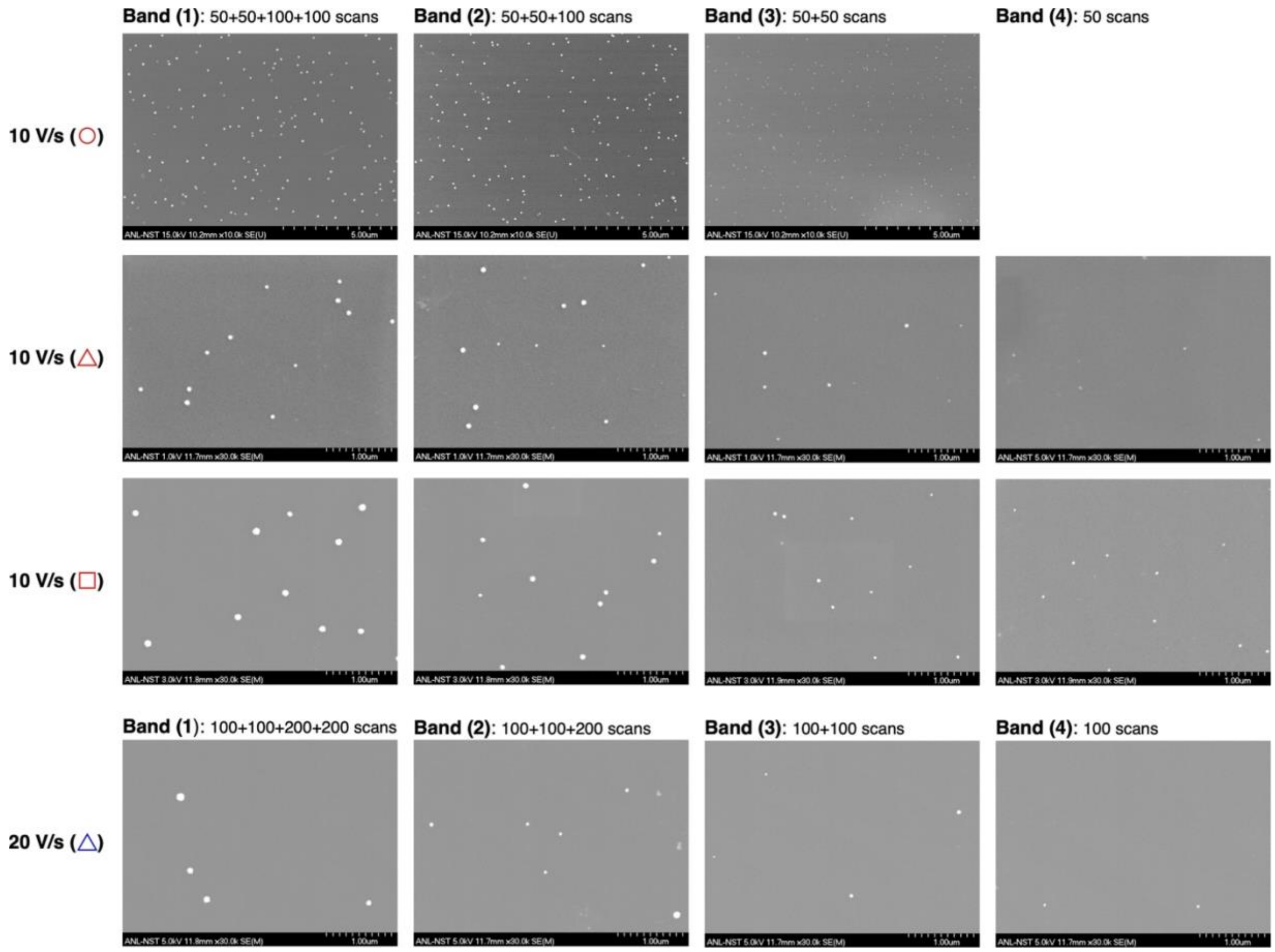

Figure S5. SEM images of the other samples that were used to obtain **Figure 3bc**. Note that the magnification of the images on the top row is ×10,000, and that of the others, including those in **Figure 3a**, is ×30,000.

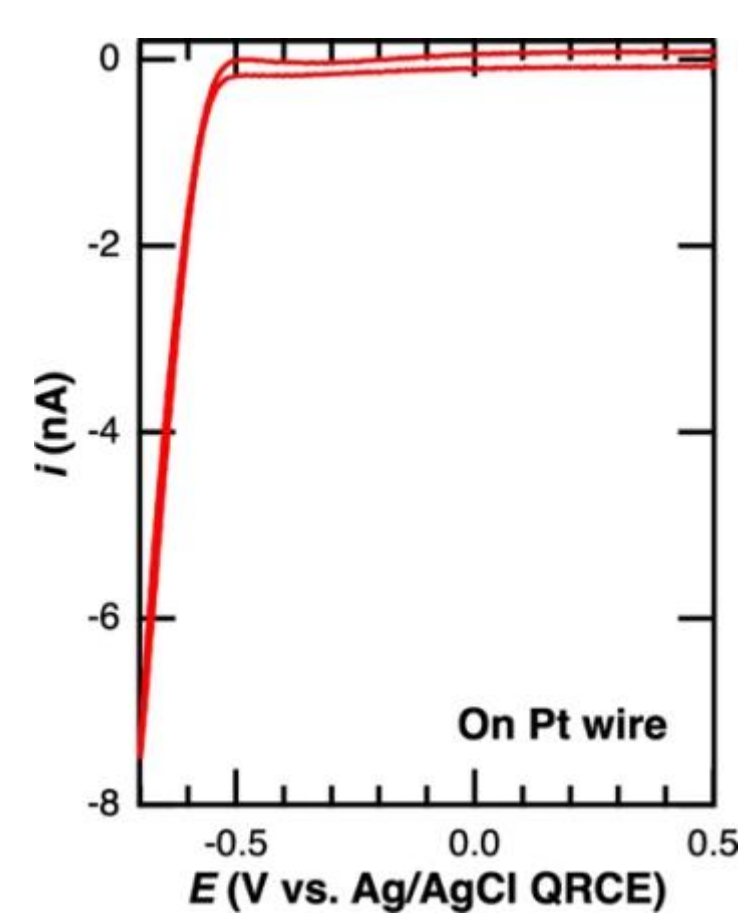

Figure S6. SECCM-CV data (scan rate: 1 V/s) averaged from 17 SECCM measurements on a Pt wire (50 μm in diameter) using a pipette (*ca*. 0.4 μm in opening diameter) in 0.1 M $HClO_4$.

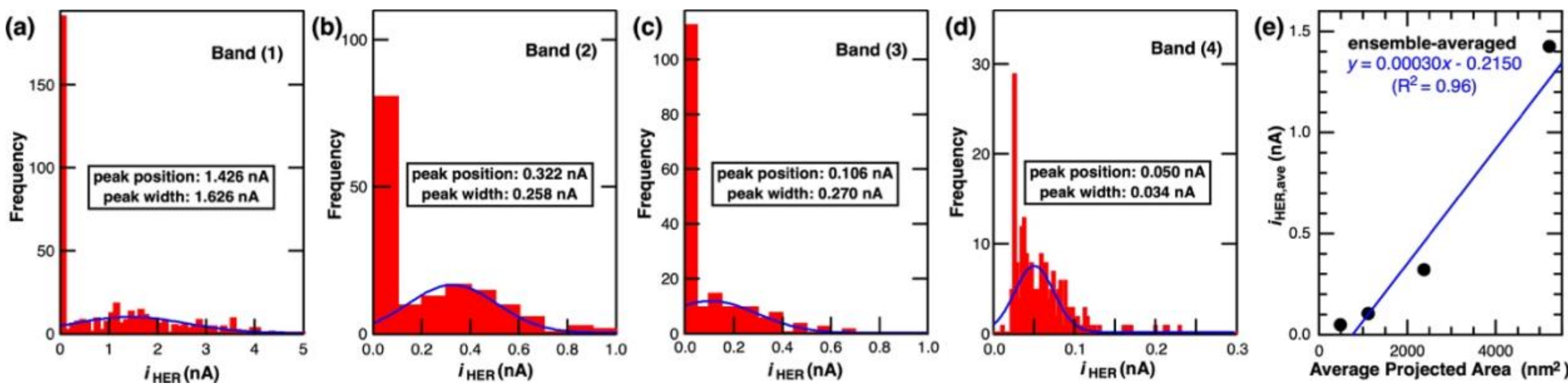

Figure S7. (a-d) A Gaussian curve obtained by fitting to the distribution of i_{HER} in each of the histograms given in **Figure 5**. Gaussian peak position (= $i_{HER,ave}$) and width are given in the inset. Each of the histograms was fitted to two Gaussian components, and only a curve fitted to the larger i_{HER} component was shown in the figure. (e) Relationship between $i_{HER,ave}$ and average projected surface area. A blue solid line was obtained by linear fitting.

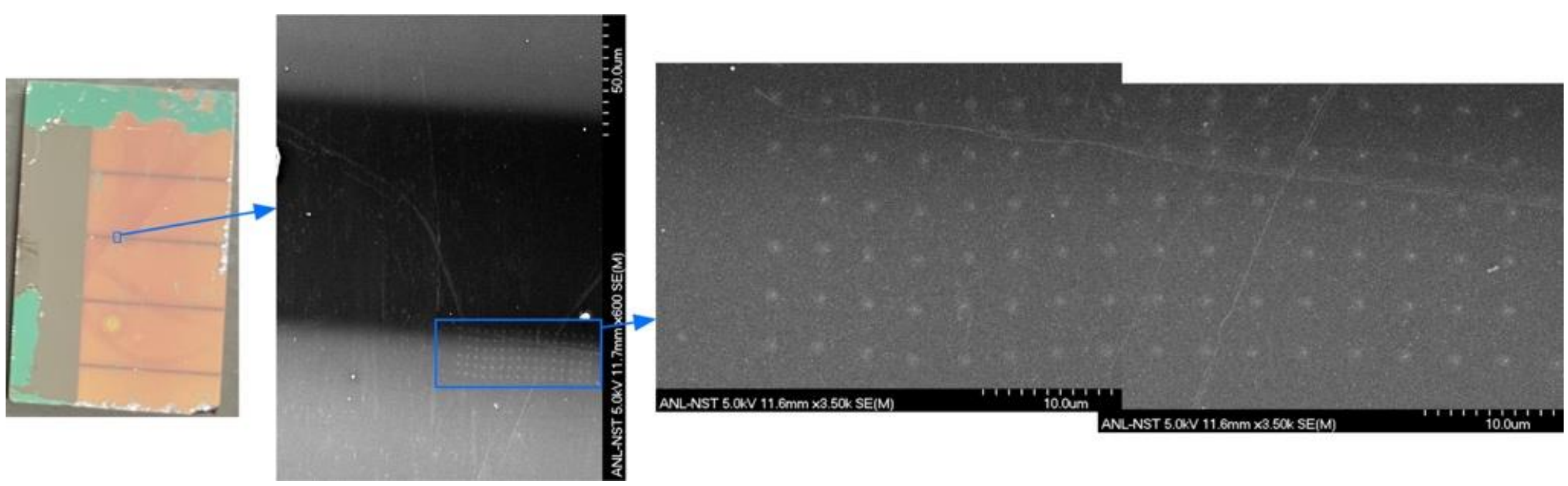

Figure S8. SEM images obtained around Band (3) on the PPF electrode (10 V/s) that show droplet footprints associated with SECCM measurements at 6 × 16 positions. Of note, the PPF electrode surface was scratched using the sharp end of tweezers to help guide the positions of SECCM measurements, as shown in the optical picture.

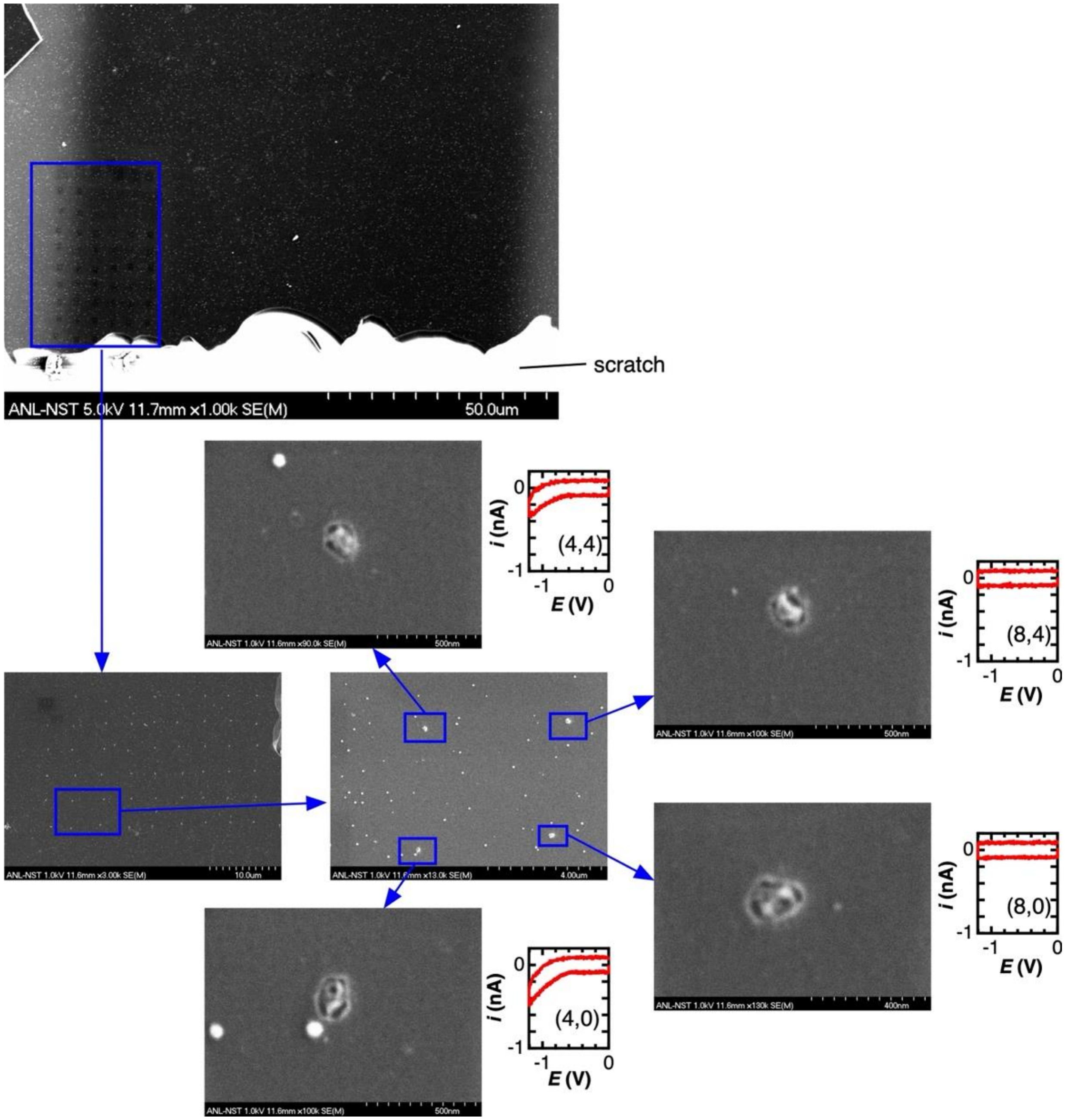

Figure S9. SEM images obtained at Band (2) on the PPF electrode (10 V/s). SECCM-CV data (scan rate: 1 V/s; in 0.1 M $HClO_4$; E (V vs. Ag/AgCl QRCE)) were measured at positions indicated shown by droplet footprints.

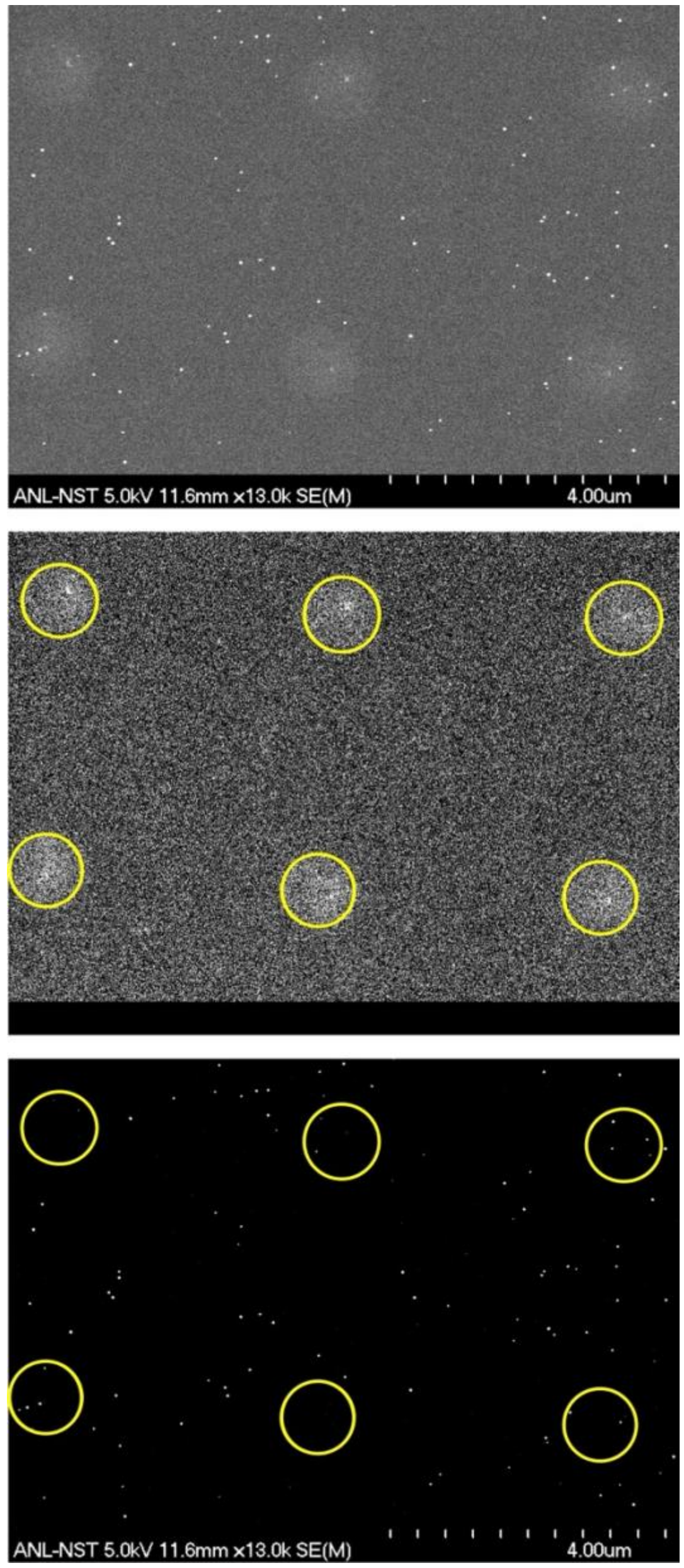

Figure S10. (Top) SEM image shown in **Figure 6d**. (Middle) The contrast of the SEM image was adjusted to highlight droplet footprints, which were shown by yellow circles. (Bottom) The contrast of the SEM image was adjusted to differentiate PtNPs from other surface structures or adsorbed impurities on the basis of their high electron scattering efficiency to determine N_{PtNP}. Note that bright dots with ≥ 4 pixels^2 in area were considered as PtNPs.
